# A Comprehensive Outlook on Pulmonary Alveolar Proteinosis—A Review

**DOI:** 10.3390/ijms25137092

**Published:** 2024-06-28

**Authors:** Julia Wołoszczak, Martyna Wrześniewska, Aleksandra Hrapkowicz, Kinga Janowska, Joanna Szydziak, Krzysztof Gomułka

**Affiliations:** 1 Student Scientific Group of Internal Medicine and Allergology, Faculty of Medicine, Wroclaw Medical University, 50-368 Wroclaw, Poland; julia.woloszczak@student.umw.edu.pl (J.W.); martyna.wrzesniewska@student.umw.edu.pl (M.W.); aleksandra.hrapkowicz@student.umw.edu.pl (A.H.); kinga.janowska@student.umw.edu.pl (K.J.); joanna.szydziak@student.umw.edu.pl (J.S.); 2 Clinical Department of Internal Medicine, Pneumology and Allergology, Faculty of Medicine, Wroclaw Medical University, 50-368 Wroclaw, Poland

**Keywords:** pulmonary alveolar proteinosis, bronchoalveolar lavage, whole-lung lavage, GM-CSF, rituximab

## Abstract

Pulmonary alveolar proteinosis (PAP) is an ultra-rare disease caused by impaired pulmonary surfactant clearance due to the dysfunction of alveolar macrophages or their signaling pathways. PAP is categorized into autoimmune, congenital, and secondary PAP, with autoimmune PAP being the most prevalent. This article aims to present a comprehensive review of PAP classification, pathogenesis, clinical presentation, diagnostics, and treatment. The literature search was conducted using the PubMed database and a total of 67 articles were selected. The PAP diagnosis is usually based on clinical symptoms, radiological imaging, and bronchoalveolar lavage, with additional GM-CSF antibody tests. The gold standard for PAP treatment is whole-lung lavage. This review presents a summary of the most recent findings concerning pulmonary alveolar proteinosis, pointing out specific features that require further investigation.

## 1. Introduction

Pulmonary alveolar proteinosis (PAP) is a rare respiratory disease that was first described by Rosen and Castleman in 1958 [1]. It is caused by the malfunction of the alveolar macrophages, leading to the accumulation of lipoproteinaceous material in the alveolar space [2].

PAP comprises a diverse range of disorders that can be classified as autoimmune, secondary, or congenital, based on their etiology and underlying pathogenesis [3]. The most common form of pulmonary alveolar proteinosis is autoimmune PAP, which accounts for 90% of all PAP cases and is characterized by the presence of anti-granulocyte-macrophage-colony-stimulating-factor (anti-GM-CSF) autoantibodies. Secondary PAP is a consequence of various diseases affecting the function or number of alveolar macrophages. It can be caused by infections, toxins, or hematological diseases, such as myelodysplastic syndromes. The congenital form of PAP is a result of mutations in the genes of surfactant proteins, lipid transporters, or alterations in lung development [2].

Although PAP may be asymptomatic, it can also present with respiratory dysfunction and lead to various clinical manifestations. The symptoms are often vague, and they include cough, dyspnea, and chest pain. However, manifestations such as weight loss, fatigue, or even respiratory failure can appear [4]. Due to the non-specific symptoms and the fact that PAP affects people in different age groups, the diagnosis may be challenging. It involves an extensive analysis that includes various forms of imaging, bronchoscopy, bronchoalveolar lavage (BAL) and GM-CSF antibody tests. In congenital and secondary forms, genetic testing is being used [2].

Treatment options vary depending on the type of the disease. Asymptomatic patients may require only monitoring, but for secondary PAP, treating the underlying cause is crucial. For symptomatic presentation, whole-lung lavage (WLL) is the most effective treatment, as it physically removes the accumulated substances from the alveolar space. Although WLL is the gold standard treatment, pharmacological therapy, including GM-CSF replacement, is still the most common approach. Other treatments, such as plasmapheresis or biological therapy, may also be used [5].

In the following research we aimed to summarize the recent findings on PAP. Our review has the objective of understanding the pathophysiology and clinical presentation of PAP, along with the current diagnostic and therapeutic approaches available for its management.

## 2. Epidemiology

PAP is classified as an ultra-rare lung disease [6]. Its prevalence varies between countries, at 3.7–40 per million citizens [7]. Most patients affected by the disease (over 90%) have autoimmune PAP, with a prevalence of 0.1/100,000; 4% constitutes secondary PAP, 1% congenital PAP, and undetermined PAP-like disease accounts for the remaining 5% [8]. Age and smoking are major risk factors for PAP development—a history of smoking occurs in 56% of patients [9] and the mean age of diagnosis is around 50 years (but it may occur in both infants and elderly people) [10,11]. Exposure to dust and vapors is another significant risk factor, predominantly associated with secondary PAP. PAP is a disease most commonly affecting men (2:1 men-to-women ratio in autoimmune PAP and 1.2:1 men-to-women ratio in secondary PAP) [7,9].

## 3. Pathogenesis

**Figure 1:**
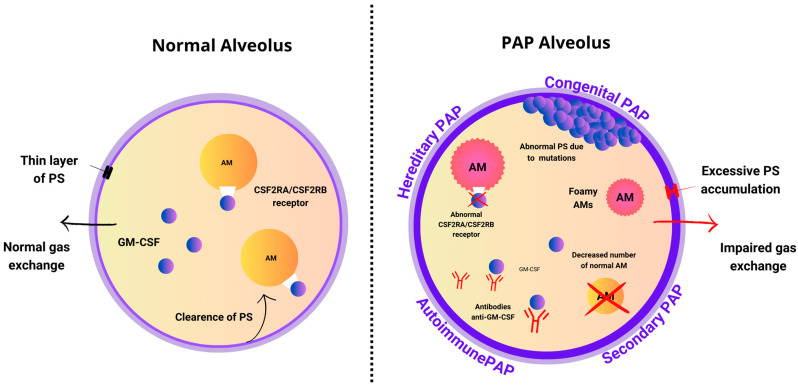
Pathomechanism of PAP—alveolar comparison.

Pulmonary surfactant (PS) is a complex consisting of polar phospholipids: dipalmitoylphosphatidylcholine (DPPC) (40%), other phospholipids (40%), and cholesterol (10%) and surfactant proteins A–D (10%). The function of PS is to prevent alveolar collapse by decreasing surface tension and protecting the lungs from infections and inflammation, acting as an opsonin as well as directly killing microbes, being an important part of host defense against microbial pathogens. PS is secreted and degraded by lung alveolar type-II (AT-II) cells, although around 30% of the surfactant is catabolized by alveolar macrophages (AMs). The balance between production and clearance enables the maintenance of surfactant homeostasis and is pivotal for the functioning of the complex [12,13]. In PAP, the alteration of surfactant homeostasis results in an accumulation of lipoproteinaceous material in pulmonary alveoli and AMs (Figure 1) [14].

Macrophages are a major group of innate immune cells that reside in various tissues, where they play essential roles as immune system sentinels. Circulating monocytes have only a partial contribution to the AMs composition, as they mostly derive from the embryonic monocyte progenitors of the yolk sac or fetal liver into the lung, where they differentiate into AMs [12,15]. AMs are capable of dividing, self-renewing, and sustaining themselves without relying on circulating monocytes. The differentiation process and expression of specific surface markers are highly influenced by the tissue environment, signaling pathways, and transcription factors [12]. The catabolism of pulmonary surfactant in alveolar macrophages is controlled by GM-CSF; therefore, disturbances of this signaling pathway will most often lead to the development of PAP.

GM-CSF is a cytokine that plays a pivotal role in the terminal differentiation of AMs, crucial for regulating innate immunity and surfactant catabolism [13]. GM-CSF binding causes the activation of Janus kinase 2 (JAK2) and the initiation of signaling via multiple pathways, including the activation of the signal transducer and activator of transcription 5 (STAT5), transcription factor PU.1 (encoded by SPI1), and the peroxisome proliferator-activated receptor-γ (PPARγ) [14]. PU.1 is critical for the differentiation of AMs and is highly dependent on GM-CSF signaling. The downregulation of PU.1 expression inhibits the transcription and expression of genes such as MR, TLR, TNF-α, Fc-γ, and IL-6/12/18. The impairment of antigen recognition, phagocytosis, pro-inflammatory signaling, and related innate and adaptive immune responses ultimately predisposes PAP patients to secondary infections [12]. GM-CSF also increases the expression and functional activity of PPAR-γ, which regulates AM developmental processes as a master transcription factor. Another vital factor of AM signaling pathways is the transforming growth factor (TGF)-β, which drives the differentiation of monocytes into AMs via autocrine and paracrine processes. GM-CSF, PPAR-γ and TGF-β-mediated signaling pathways are critical for AM development [16,17].

AMs display both immunosuppressive and pro-inflammatory functions, depending on the alveolar microenvironment. Immunosuppressive activity is favored in non-inflammatory microenvironments, while pro-inflammatory functions are induced in inflammatory microenvironments. Under normal conditions, AMs produce IL-10, TGF-β, and CD200, but pro-inflammatory activity is triggered by TLR signals and NETs. To maintain homeostasis, the process of efferocytosis degrades apoptotic and necrotic cells that may otherwise induce pro-inflammatory responses [17].

The disruption of the GM-CSF→PU.1→PPARg signaling axis reduces the expression of a critical macrophage lipid exporter, ABCG1 [18]. The GM-CSF–PU.1–PPARγ–ABCG1 axis in alveolar macrophages is crucial for the maintenance of surfactant homeostasis through lipid clearance. GM-CSF regulates cholesterol efflux from macrophages; it is not obligatory for the degradation of phospholipids. The disruption of GM-CSF signaling causes the primary disturbance in lipid metabolism in alveolar macrophages. The lipids, taken up by AMs, are degraded into free cholesterol and free fatty acids in lysosomes [19]. Subsequently, the free cholesterol is re-esterified into cholesterol esters in the endoplasmic reticulum of the cell, and stored in the cytoplasm as lipid droplets. The reduction in surfactant clearance is a secondary consequence of the reduced surfactant uptake by foamy, overloaded AMs. The loss of GM-CSF signaling in PAP is associated with a marked increase in the cholesterol: phospholipid ratio in pulmonary alveolar surfactant, which is relevant to surfactant function. Research on lipidomic in PAP shows that total lipid concentration in the alveolar airspaces increased 12- to 59-fold in alveolar fluid from PAP patients compared with healthy individuals. Changes in lipogram varied due to lipid classes—free cholesterol was increased 60-fold, cholesteryl esters were increased 24-fold, and total phospholipids were increased 19-fold [20].

In the light microscopy, PAP is presented with characteristic features: diastase-resistant PAS-positive intracellular inclusions, creating the impression of ‘foamy’ macrophages; acellular globules; basophilic on May–Grunwald–Giemsa staining; pink with PAS staining, but without alcian blue staining, which differentiates them from mucins; and large amounts of amorphous debris, representing myelin-like multilamellated structures and lamellar bodies, which, due to weak PAS staining, create the impression of the characteristic ‘dirty background’ [21].

## 4. Classification

PAP, as a syndrome of abnormal PS homeostasis, is divided into three types: autoimmune PAP, formerly counted along with hereditary PAP as idiopathic or primary PAP, congenital PAP, and secondary PAP, distinguished due to their pathomechanism and underlying causes (Table 1) [13]. There is a subset of PAP patients who do not meet the forementioned criteria of each type, and the etiology of disease is uncertain. This group represents the unclassified type of PAP.

### 4.1. Autoimmune PAP

Autoimmune PAP occurs when the dysfunction of GM-CSF signaling induces an abnormal macrophage and neutrophil activation, which leads to impaired PS homeostasis and the further accumulation of surfactant. Such dysfunction is a result of the GM-CSF neutralizing antibodies present in the serum and bronchoalveolar lavage fluid. Polyclonal immunoglobulin G blocks GM-CSF signaling in vivo through targeting multiple epitopes of GM-CSF molecules. This demonstration may suggest that there are also various clones of autoantibodies responsible for the pathogenesis in each patient. Inhibited biological activity of GM-CSF disables the AMs to degrade surfactant, reduces the effectiveness of gas exchange as well as impaires basal and antimicrobial functions. It might also be a trigger for the development of pulmonary fibrosis [3,13]. There has been no correlation observed between the disease severity score (DSS) and levels of anti-GM-CSF autoantibody in the serum [9,22].

Some recent studies presented a spectrum of possible molecular variations related to aPAP mechanisms, one of which was the association with HLA, as a shared feature among many autoimmune diseases. A study based on a small size sample of 47 Caucasian patients in the USA has shown no existing link between aPAP and HLA variability [23]. Another study with 198 aPAP patients of Japanese descent shows a strong genetic risk identified within the MHC region. Research suggested HLA-DRB1*08:03 to be the lead HLA allele increasing the risk of aPAP, associated with the increased production of anti-GM-CSF antibodies. In this situation, while a small sample size can potentially affect the result, it is vital to also consider the selection of the population. The HLA-DRB1*08:03 is prevalent among Asians; however, it is scarce among Caucasians and Europeans in the USA, which is the reason for the significant discrepancy in the results [24]. In other research, the relation between aPAP and the high concentration of B cell-activating factor (BAFF) and proliferation-inducing ligand (APRIL) was confirmed. BAFF and APRIL play a part in the selection, activation, maturation, and survival of B cells, also being expressed in monocytes/macrophages, dendritic cells, and activated T lymphocytes [25]. The same cytokines’ levels were elevated in other autoimmune diseases, among them systemic lupus erythematosus (SLE), Sjögren’s syndrome (SjS), rheumatoid arthritis, and immunoglobulin G4 (IgG4)-related disease [26,27,28,29]. BAFF and APRIL levels of sera and BALF in aPAP were significantly increased compared with healthy volunteers and disease control, and correlated with DSS [25].

### 4.2. Hereditary PAP

Hereditary PAP (hPAP) is a genetic disease that accounts for only 3% of all PAP syndromes. It is caused by autosomal recessive mutations in CSF2RA or CSF2RB in the GM-CSF receptor α or β chains, that lead to impaired macrophage maturation. The GM-CSF receptor β chain was suggested to be critical in surfactant homeostasis in humans and in the pathogenesis of cPAP [30]. It results in the dysfunction of AM receptors that interact with GM-CSF. GM-CSF antibody levels are not elevated [1]. Historically, hPAP and aPAP were classified as one type—idiopathic PAP, as they present the same clinical and histological picture [5]. In 2017, one report presented the case of a 77 year old female patient with late-onset congenital PAP associated with a genetic defect in CSF2RA, disabling GM-CSF signaling. The results suggested the existence of compensation mechanisms from other signaling pathways, which may lead to a postponed onset presentation of cPAP [31].

### 4.3. Congenital PAP

Congenital PAP accounts for 1.5% of PAP syndromes. It is a disease of the newborn, but is rarely also seen in adults [5]. Mutations concern surfactant proteins B or C (SFTB, SFTC), ATP-binding cassette (ABCA3), subfamily A member 3 (ABCA3), and thyroid transcription factor TTF1 (NKX2-1)-1 [13,14,32]. GM-CSF autoantibodies were also present in healthy donors, although far lower than those present in PAP patients. These results suggested the hypothesis of a high risk of PAP correlated with GM-CSF autoantibody levels increased above a critical threshold, which has been reported to be ~5 μg/mL [30,33]. Reduced protein expression on the cell surface disables GM-CSF signaling. cPAP typically manifests in neonates, although it has variable courses regarding individual mutations. Infants homozygous for recessive loss-of-function mutations in SFTPB develop respiratory failure and die shortly after birth [13,14]. Individuals heterozygous for recessive loss-of-function SFTPB alleles present normal lung function. Patients with autosomal dominant mutations in SFTPC can develop interstitial lung disease regardless of age group. Homozygous infants for recessive loss-of-function mutations in ABCA3 suffer from fatal surfactant deficiency, and also they present higher risk of premature death. Other ABCA3 mutations result in dysfunctional PS deficiency in phosphatidylcholine, causing chronic respiratory disease in older children and adults. The haploinsufficiency of TTF1 may result in a complex phenotype in neonates, including hypothyroidism, brain abnormalities and acute and chronic lung disease [3]. Often, concomitant pulmonary fibrosis can also cause PAP [13].

### 4.4. Secondary PAP

Secondary PAP (sPAP) is defined clinically as the occurrence of the syndrome with an underlying disease known to be associated with the development of PAP. Elevated GM-CSF autoantibody levels are not observed—the diagnosis is usually made based on the presence of typical radiological manifestations and lung histopathological findings.

The pathogenesis of sPAP is still poorly understood [30]. It is thought that it is caused by a reduction in either the functional capacity or absolute numbers of AM, but data are still limited [18]. AM dysfunction may be triggered mainly by hematological or environmental factors, but also by solid malignancies, infectious diseases, autoimmune diseases, and medications. sPAP may be associated with deficiency syndromes, including thymic alymphoplasia, immunoglobulin A deficiency, AIDS and immunosuppression following solid organ transplantation. Another important factor is occupational exposure to dust and toxic inhalation, such as: inorganic and organic dust, among them silica, cement, titanium, aluminum, sawdust, fertilizer, bakery flour, and fumes [30,34,35]. The presentation of sPAP can be fulminant and has a poor prognosis compared to aPAP [13,14,36].

The pathomechanisms of sPAP are still unclear, although researchers have described some of the mechanisms, mostly those related to hematological diseases. There are suggestions that sPAP may be caused by an acquired loss of GM-CSF signaling, reduced AM numbers, or AM dysfunction [30]. Iriguchi, in research on mice models, reported a link between the development of PAP and T-bet (master transcription factor in type-1 helper T lymphocytes) overexpression in T lymphocytes associated with hematological disorders. T-bet may cause mastocytosis with a massive infiltration to alveoli, at the same time spontaneously inducing maturation arrest in the mononuclear phagocyte lineage, leading to a slowdown of surfactant clearance [37]. In a 2019 case report, sPAP was diagnosed in a 77-year old patient during the treatment of atypical chronic myeloid leukemia (aCML).Due to undergoing organizing pneumonia associated with aCML, prednisolone was administered, without improvement in the patient’s condition. Although the cause of sPAP development was not clear, steroids were suspected triggers, as they are known for increasing the production of phospholipids and depressing the monocyte function [38]. Another pathomechanism was suspected in patients diagnosed with sPAP after lung transplantation. Recurrent pulmonary infections with Aspergillus fumigatus and Mycobacterium intracellulare, among other pathogens, have aroused suspicion of AM dysfunction. Although the first theory was immunosuppressant-related sPAP, it was assumed that the main cause of sPAP development was oxidative stress triggered by ischemia-reperfusion at the time of transplantation or infection. It led to the accumulation of lipofuscin, conducive to a subsequent triggering of inflammation and oxidative stress [39].

## 5. Clinical Presentation

PAP presents a wide spectrum of onsets, ranging from spontaneous resolution to progressive respiratory failure or death from infections. The symptoms of PAP are nonspecific. Between 50 and 90% of patients with PAP report progressive dyspnoea, mostly productive cough and other symptoms, including fatigue, weight loss, chest discomfort, arthralgias, and fever. The fever may be the direct symptom of PAP or it could be also related to secondary infections [13].

The course of PAP depends highly on its type. Individuals with aPAP usually present in the third to fourth decades of life, with progressive dyspnea of insidious onset [30]. hPAP presents a course similar to aPAP, but in late infancy or childhood. Patients with secondary PAP present in the context of underlying environmental exposure or other underlying clinical conditions [14]. The presentation of cPAP depends mostly on mutation types. In newborns, this usually includes respiratory distress, proceeding rapidly to intubation and mechanical ventilation. In older children, the first symptoms are usually cough, progressive breathlessness and respiratory distress, although it may occur with type I respiratory failure, requiring intubation [40].

Pulmonary function tests in PAP patients are non-specific, both in adults and in the pediatric population. Restrictions appear in advanced disease, especially with the development of fibrosis. Disease severity, in terms of the presence of symptoms and degree of hypoxemia, correlates with a disproportionate reduction in carbon monoxide diffusion capacity (DLCO) and a moderate decrease in functional volumes. Hypoxemia is present initially upon exercise and, later in the course of the disease, also at rest [2,5,39]. Eventually, in adults with aPAP, hypoxemia at rest was present in approximately one-third, and around 50% of the patients presented this symptom during exercise, while in the pediatric population presentation, 55% of the patients with hPAP had hypoxemia [41].

A crucial pathomechanism of PAP is an impairment of the innate immune functions of myeloid cells, which results in reduced phagocytosis, microbial killing, proinflammatory signaling. The effect of those dysfunctions is a susceptibility to opportunistic infections, associated with poor prognosis [42], as it occurs in 13% of cases and accounts for 18–20% of deaths related to PAP. Usually identified within 16 months of PAP diagnosis, infections can be pulmonary as well as extrapulmonary [14]. It may indicate that the immune dysfunction and predisposition to infection observed in aPAP occur at the systemic level, and are not limited to only the respiratory system [43]. Opportunistic pathogens include *Nocardia*, *Mycobacterium*, *Aspergillus*, *Streptococcus*, *Pseudomonas*, *Pneumocystis*, *Adenovirus* and fungal species [18,30].

An assessment of the severity of the disease’s course is possible by using the scoring systems Severity and Prognosis Score of PAP (SPSP) and Disease Severity Score (DSS). DSS, which is based on the presence of symptoms and the degree of reduction in PaO2, was suggested as an index of the severity of PAP and was divided into 5 grades. SPSP evaluates smoking status, symptoms, PaO2, HRCT score, and DLCO, ranging these parameters from 1 to 10. It presents stronger correlations with FVC, FEV1, DLCO, and HRCT scores than DSS, at the same time better illustrating the severity of the course. Both scales are reliable in assessing disease severity, yet predicting disease progression or relapse value is limited [5,44].

## 6. Diagnostics

The differential diagnosis of PAP syndrome is challenged by its low prevalence and nonspecific symptoms; PAP often gets misdiagnosed as pneumonia at first. After multiple rounds of ineffective empiric antibiotics, doctors start reconsidering the diagnosis and referring the patient for further evaluation. This delay in reaching an accurate diagnosis typically averages about 1.5 years [45]. The clinical and radiological features of PAP overlap with other respiratory conditions, such as sarcoidosis, Langerhans cell histiocytosis, and hypersensitivity pneumonitis [46]. PAP also requires a thorough differential diagnosis for connective tissue disease-related interstitial lung disease (CTD-ILD) [47,48]. Around one-third of patients with PAP do not manifest any symptoms, and those who do often experience non-specific ones. Dyspnea is the most common, followed by chronic cough and bronchitis. Symptoms like chest pain and hemoptysis are rare and could indicate concomitant infections. Systemic symptoms, such as fatigue and weight loss, often present for months before evaluation, and may also point towards PAP. During clinical examination, in auscultation, crackles are present in about half of cases. Digital clubbing is uncommon and present in only about 20% of cases [2]. Lactate dehydrogenase (LDH), a non-specific serum biomarker, is elevated in up to 80% of PAP patients [10]. Some PAP patients may present with slightly elevated C-reactive protein (CRP) and erythrocyte sedimentation rate (ESR), lowered arterial oxygen pressure (below 70 mm Hg), or an increase in carcinoembryonic antigen (CEA) levels [49].

**Figure 2:**
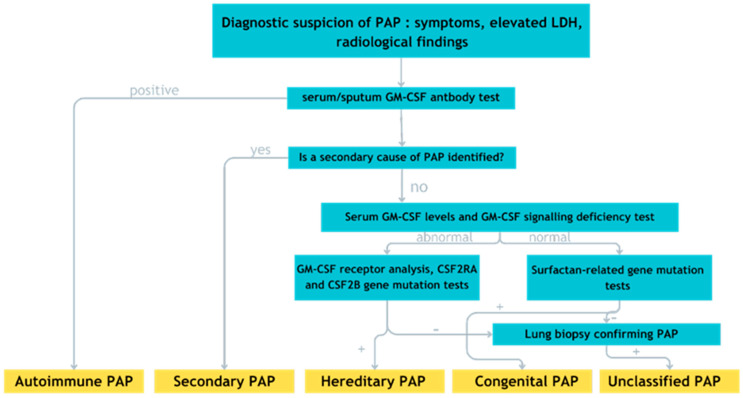
Algorithm for primary alveolar proteinosis diagnosis. The plus (+) sign indicates a positive test result, and the minus (−) sign indicates a negative test result.

PAP diagnosis typically involves analyzing a coexistence of symptoms, incorporating detailed imaging like HRCT scans, and using bronchoscopy to examine the BALF with Periodic Acid–Schiff (PAS) staining. Sometimes, a biopsy of lung tissue might also be required during bronchoscopy. Additionally, testing for autoantibodies against GM-CSF in either BALF or blood serum is part of the diagnostic process. This extensive approach helps doctors accurately identify PAP and understand it (Figure 2) [50].

### 6.1. Imaging

On chest X-ray, it is common to observe diffuse symmetrical bilateral alveolar opacities in a perihilar and basilar distribution, which can progress to confluent infiltrates involving all the five lobes, sometimes referred to as a “batwing” or “butterfly wing” distribution. It may demonstrate without air-bronchograms or resemble pulmonary edema without cardiomegaly or pleural effusions [7].

Chest CT, specifically high-resolution CT (HRCT), plays a pivotal role in PAP diagnosis; it reveals intralobular thickening and diffuse ground-glass opacities, creating a “crazy-paving” pattern. The “crazy paving” is highly characteristic, but not pathognomonic, for PAP [5]. Typically, these changes are distributed in patches or in a geographical pattern (healthy and affected lung regions are juxtaposed or interspersed) [2]. Repeated CT scans of the same patient often show fluctuations over time, indicating that PAP can either spontaneously improve or deteriorate, even with treatment [5].

### 6.2. Bronchoscopy and BAL

Bronchoscopy with bronchoalveolar lavage (BAL) is a minimally invasive technique, a first step and a gold standard in the diagnostic and prognostic evaluation of parenchymal lung diseases [51]. It can be used to diagnose PAP; however, it will not identify its cause [14]. In PAP patients, bronchoalveolar lavage fluid (BALF) has a milky and opaque appearance, due to the high content of lipoproteinaceous material. It typically settles into two fractions—a cloudy sediment layer with a translucent supernatant. In a microscopic examination, one can detect foamy, enlarged macrophages with eosinophilic granules inside, alongside extracellular hyaline, positive with PAS staining and negative with Alcian blue staining. Abundant, myelin-like lamellar bodies with concentric laminations can be discovered in electron microscopy [5,52]. Additionally, it is essential to rule out fungal, mycobacterial, and other infectious causes using specific stains and microbial cultures [14]. Sometimes, when BALF is inconclusive, obtaining a biopsy might be necessary; transbronchial biopsy is performed more often than open-lung biopsy [9].

### 6.3. GM-CSF Antibody Test

Detecting antibodies against GM-CSF in circulation, specific to autoimmune PAP, aids in distinguishing it from other forms of the condition. A gold standard, ELISA, or latex agglutination test, using latex beads coupled with recombinant human GM-CSF, are the main methods used to detect GM-CSF in a patient’s serum [50]. In patients with autoimmune PAP, the serum levels of GM-CSF antibodies are elevated, exceeding >5 μg·mL^−1^, whereas healthy individuals and those with secondary or congenital PAP usually exhibit low levels [3,53]. The serum concentration of these antibodies does not correlate with disease severity [2].

When GM-CSF autoantibodies are absent or found in low levels in patients diagnosed with PAP without a clear secondary cause, further tests like GM-CSF concentration and signaling tests are required. Serum GM-CSF concentration testing is recommended for PAP patients with normal GM-CSF autoantibody levels, especially after excluding secondary PAP. Elevated serum GM-CSF concentration (>10 pg/mL^−1^) indicates hereditary PAP caused by CSF2RA or CSF2RB mutations. Assessing GM-CSF signaling includes examining the activation of intracellular phosphorylated STAT5, or the presence of cell-surface CD11b in neutrophils when stimulated by GM-CSF. If the results are positive, it suggests the need for further gene analysis to identify mutations in CSF2RA or CSF2RB [3].

### 6.4. Genetic Testing for Causes of Congenital and Secondary PAP

Genetic tests in PAP should include screening for mutations in the genes taking part in surfactant production—SFTPA, SFTPB, SFTPC, ABCA3, or TTF1. While mutations in SFTPB and ABCA3 cause a significant disruption in surfactant production and respiratory failure in newborn infants, mutations in SFTPC and SFTPA usually clinically emerge later, in infancy or in adulthood [3]. Genes associated with the development of secondary PAP include SLC7A7 and MARS [14].

## 7. Treatment

The treatment strategy for PAP depends on the specific type of the condition and disease advancement level. Since PAP was discovered in 1958, various treatment approaches have been investigated, such as antibiotics, corticosteroids, potassium iodide for dissolution, streptokinase, trypsin, heparin, acetylcysteine, and several others [54]. Current management varies from monitoring patients with asymptomatic autoimmune PAP to employing whole-lung lavage (WLL) therapy, which is considered the gold standard and the most effective treatment at present [3]. In cases of secondary PAP, addressing the underlying condition is crucial [5].

### 7.1. Whole-Lung Lavage

WLL, the favored approach for PAP, involves the physical removal of the lipoproteinaceous substance from the alveolar spaces, effectively reversing the physiological defects. The indication for considering WLL is difficulty with daily tasks due to dyspnea. Some researchers suggest that patients with a PaO2 below 70 mmHg when breathing room air, or an alveolar-arterial [A-a] oxygen gradient exceeding 40 mmHg, are more likely to deteriorate and therefore may benefit from WLL. While the specific criteria for WLL indications vary between different medical centers, common reasons cited for the procedure include declining lung function, declining oxygen levels, and deteriorating radiographic findings [55]. There is no standardized protocol for conducting WLL due to the rareness of the condition, which makes it difficult to conduct randomized trials or thoroughly assess the effectiveness of specific procedural aspects. The time gap between the initial lavage and the subsequent session varies across different medical centers, with approximately half of them opting for a 1- to 2-week interval [3,54].

The whole-lung lavage (WLL) procedure, conducted under general anesthesia in an operating room, involves several steps. The patient is intubated with a double-lumen endotracheal tube (DL-ETT), and fiberoptic bronchoscopy is utilized to confirm the correct tube placement. The patient is in the lateral decubitus position, and as mechanical ventilation supports one lung, the opposite lung undergoes a cyclical process of being filled with 500–1000 mL of warmed saline (temperature of 37 °C) and is subsequently emptied through gravity drainage. Manual chest percussion can assist in enhancing drainage. The lavage process persists until the drained fluid appears clear, which may require several hours and the infusion of 15–20 L of saline solution for a single lung. After WLL completion, two-lung ventilation is reestablished, and the double-lumen endotracheal tube DL-ETT is replaced with a single-lumen tube. Afterward, patients are moved to either the recovery room or the respiratory care unit. Depending on their blood circulation and gas exchange, some patients may be extubated on the same day [56,57].

Whole-lung lavage is thought to be a safe and effective way of treating PAP. In an article by Kaenmuang P. and Navasakulpong A., performing WLL in PAP patients resulted in significant improvements in their SpO2 level (from 86% to 94%), PaO2 level (from 49.3 to 66.1), and the mMRC dyspnea score (from 3 to 2) [58]. WLL increases the chances of survival significantly, with more than 70% of patients remaining without recurring PAP for over seven years [59].

### 7.2. GM-CSF Replacement Therapy

While WLL remains a gold standard treatment in primary alveolar proteinosis, in autoimmune PAP, drug therapy is predominantly recommended. Two methods of administering recombinant GM-CSF (rGM-CSF) treatment (molgramostim and sargramostim), through inhalation and subcutaneous injection, have been tested in patients with autoimmune PAP [5]. The aim of administering external GM-CSF is to ensure there is enough GM-CSF to counteract the GM-CSF autoantibodies and replenish the depleted natural growth factor [60].

The first form of GM-CSF therapy was subcutaneous administration [3]. In follow-up studies on patients with autoimmune PAP, who received increasing doses of subcutaneous GM-CSF for 3 to 6–12 months, 43% and 48% of them responded positively, respectively. Similar results were seen in other case studies, where around half of the patients showed clear improvements [14]. A study by Zhang F. et al. supported the theory that administering low doses (75 μg–150 μg per day) of rhGM-CSF via subcutaneous injection proves to be an effective treatment for individuals with idiopathic PAP [61].

Nebulized GM-CSF is a promising therapy in autoimmune PAP. Inhaling GM-CSF directly targets the lungs, potentially leading to a greater deposition of GM-CSF in the alveoli compared to subcutaneous injection. This targeted approach could yield more effective results in PAP treatment [5]. A study by Luisetti, M. et al. demonstrated that rGM-CSF aerosolized with a highly efficient nebulizer, the AKITA2 APIXNEB^®^, resulted in the highly effective lung deposition of the rGM-CSF formulation in vitro. Furthermore, the biological activity of aerosolized rGM-CSF was completely preserved after the aerosolization process. Aerosolized rGM-CSF is likely to produce positive results when used for in vivo delivery to the airways of autoimmune PAP patients [62]. Aerosolized GM-CSF (sargramostim) shows good tolerance and safety in trials. Inhaled recombinant human GM-CSF treatment correlates with patient modest improvement in dyspnea and six-minute walk distance [63]. In a multicenter, double-blind trial, 64 patients with mild to moderate PAP were treated with inhaled GM-CSF (sargramostim). When compared to a placebo, inhaling GM-CSF led to a statistically significant, yet slight enhancement in the alveolar-arterial oxygen gradient. However, there were no significant clinical improvements observed in outcomes [64]. In a combined analysis of 10 observational studies by Sheng et al., involving 115 autoimmune PAP patients, GM-CSF exhibited an 80% response rate with a 22% relapse rate and enhanced oxygenation indices. Inhaled GM-CSF therapy showed higher response rates and improvements in A-a gradient and PaO2 compared to subcutaneous GM-CSF treatment, indicating potential benefits for autoimmune PAP patients. [65]. WLL with GM-CSF replacement therapy holds promise as a combined therapy for aPAP. A long-term, prospective, randomized trial by Campo, I et al. showed that inhaling rGM-CSF (sargramostim) following WLL reduced the need for WLL, improved pulmonary gas exchange, diminished the pulmonary surfactant accumulation and decreased the levels of serum aPAP biomarkers. Additionally, it was well tolerated, deemed safe, and did not lead to more frequent adverse events compared to WLL alone [66].

### 7.3. Plasmapheresis

While systemic anti-GM-CSF antibodies are present in autoimmune PAP patients, plasmapheresis could be a possible therapeutic option for disease management. Plasmapheresis holds promise in lowering the levels of circulating antibodies, potentially allowing for the restoration of surfactant balance [5]. However, there is limited supporting evidence for the use of plasmapheresis in PAP patients, since its use has been described in few case reports, with varied results [67,68,69].

### 7.4. Biological Therapy—Rituximab

Rituximab is a promising strategy for PAP management. It is a monoclonal antibody directed against the CD20 antigen on B lymphocytes, proven to be an effective treatment for various hematological and autoimmune disorders [5] In autoimmune conditions, reducing B cell numbers leads to a decrease in antigen-presenting B cells. This process impacts T-cell activation and reduces cytokine production, ultimately resulting in fewer plasma cells producing antibodies, like GM-CSF auto-antibodies [50]. In a study by Kavuru M S et al., 10 PAP patients were given 1000 mg intravenous rituximab infusions 15 days apart. Those patients showed improvement in arterial blood oxygenation, lung function, and high-resolution computed tomography scans [70]. In a case study by Bird D., a 41-year-old man with autoimmune PAP, in whom further WLL was contraindicated, received rituximab treatment. Within six months, a notable improvement in his clinical response was observed; this included enhancements in arterial oxygenation, respiratory membrane gas diffusion, a six-minute walk test, and radiological findings [71]. In a case study by Amital A. et al., a woman with deteriorating PAP dramatically improved after rituximab therapy; her oxygen saturation on room air rose to 98% during exercise, eliminating the need for supplemental oxygen. The capacity for carbon monoxide diffusion increased from 27% to 48% of the expected value, and there were noticeable improvements in her chest X-rays [72].

To conclude, rituximab demonstrates encouraging outcomes in the majority of patients who receive it. Adverse reactions to rituximab among PAP patients are rare and minor, and include fatigue, headaches, dizziness, nausea, decreased appetite, nasal congestion, upper respiratory infections, and chest pain [50]. However, currently only one study by Soyez, B. et al. reports the side effects of rituximab treatment after 12 months of therapy, and there are no randomized controlled trials discussing this issue [73]. Therefore, more research is necessary to investigate the potential adverse effects of rituximab treatment in PAP patients.

### 7.5. Targeting Lipids

In individuals with PAP, alveolar macrophages exhibit a significant rise in cholesterol levels (which regulates surfactant fluidity) but only a slight increase in phospholipids. Consequently, the pulmonary surfactant displays an elevated ratio of cholesterol to phospholipids. Oral statin therapy in PAP patients has been linked to improvements in clinical, physiological, and radiological outcomes [74]. In a study by Shi S et al., PAP patients without hypercholesterolemia received 12 months of statin therapy, which resulted in improvements in arterial blood gas measurement, pulmonary function, and radiographic assessment [75].

## 8. Conclusions

Pulmonary alveolar proteinosis (PAP) is a syndrome characterized by surfactant clearance disorders which presents a broad spectrum of symptoms, a diverse course, and a complex origin. While most cases result from autoimmune PAP, secondary PAP poses a greater diagnostic challenge, given the variety of unclear etiologies of the pathomechanism responsible for its development. The scarcity of the syndrome and nonspecific symptoms, coupled with opportunistic infections, may impede its identification; however, innovative diagnostic tools have made it possible to initiate the therapeutic process rapidly. The diagnosis of PAP consists of clinical symptoms, radiographic findings in X-ray or HRCT, bronchoalveolar lavage, GM-CSF antibodies tests, and genetic tests. In some cases, lung biopsy might be performed. The gold standard treatment of PAP is whole-lung lavage; in some patients, subcutaneous or inhaled GM-CSF replacement therapy, or plasmapheresis, is advised. Rituximab and statins are pharmacological options showing therapeutic promise in the future management of PAP. However, they require further investigation.

## Figures and Tables

**Table 1:** Types and causes of PAP.

Types	Autoimmune PAP/Hereditary PAP (Primary PAP)	Congenital PAP	Secondary PAP
Causes	Autoimmune PAP (GM-CSF antibody+) Hereditary PAP (GM-CSF antibody−)	SFTPC (SP-B), SFTPC (SP-C) mutation ABCA3 mutation TTF1 mutation	Hematological disorders Chronic infections Chronic inflammation Immune deficiencies and dysregulation, lung transplant, bone marrow transplant Drug-induced, including chemotherapy.

## Data Availability

Data sharing is not applicable as no datasets were generated or analyzed during the current study.
